# Whole-genome sequence of *Proteus faecis* CR112, isolated from the gut of a healthy *Labeo rohita*

**DOI:** 10.1128/MRA.00953-23

**Published:** 2023-12-19

**Authors:** Sulav Indra Paul, Saif Uddin Khan, Md Javed Foysal, Md Mahbubur Rahman

**Affiliations:** 1 Institute of Biotechnology and Genetic Engineering, Bangabandhu Sheikh Mujibur Rahman Agricultural University, Gazipur, Bangladesh; 2 Department of Genetic Engineering and Biotechnology, Shahjalal University of Science and Technology, Sylhet, Bangladesh; University of Maryland School of Medicine, Baltimore, Maryland, USA

**Keywords:** antibacterial activity, *Proteus faecis*, *Labeo rohita*

## Abstract

Bacteria of the genus *Proteus* are gram-negative bacilli. *Proteus faecis* CR112 was isolated from fish gut based on its antibacterial activity against fish pathogens. The genome sequence of CR112 provided valuable information about its secondary metabolite gene clusters.

## ANNOUNCEMENT

*Proteus* species are widespread in aquatic environments and are known to be associated with fish and shrimp diseases (1). However, till now, no report has been found about the antibacterial activities of *Proteus faecis* against fish pathogens.

To isolate strain CR112, the abdomen of a healthy *Labeo rohita* specimen was cut aseptically, and the gut was taken out. One-gram homogenates of the intestinal segments were serially diluted and spread onto de Man, Rogosa, and Sharpe (MRS) agar plates and incubated at 28°C for 48 h. CR112 was picked from the growing colonies on the MRS plate. This strain exhibited *in vitro* antagonistic activities against highly virulent fish pathogenic *Aeromonas veronii* and *Enterococcus faecalis* strains and prevented motile *Aeromonas* septicemia in *L. rohita* (2) and *Oreochromis niloticus* (3), as well as streptococcosis in *O. niloticus* (3). The animal experiments were granted ethical approval by the Institute of Biotechnology and Genetic Engineering (IBGE) ethical review committee (ERC), with the approval number IBGE-ERC-008.

To obtain the genomic DNA from CR112, a single colony from the original stock was grown in de Man, Rogosa, and Sharpe broth for 48 h at 28°C. Then, the DNA was extracted from the broth using a GeneJET Genomic DNA Purification Kit (Thermo Fisher Scientific, USA) following manufacturer’s instructions. The purity and quantity of the extracted DNA were assessed using the QuantiFluor ONE Double-Stranded DNA Kit (Promega, USA). A paired-end DNA library was prepared using Nextera XT Library Prep Kit (Illumina, San Diego, CA, USA) following manufacturer’s instructions (4). Genome sequencing (600 cycles) was performed using the Illumina MiSeq benchtop sequencer (5), yielding a total of 2,223,208 paired-end reads with 1,046,631,911 bases. The quality of the raw reads was determined using PRINSEQ v.0.20.3 (6), and low-quality sequences were trimmed using Trimmomatic v.0.38 (7). The resulting high-quality reads were assembled into draft genomes using SPAdes v.3.9.0 (8). QUAST v.5.0.2 (9) was used for the quality assessment of the assembled genome. Genome annotation was performed using the NCBI Prokaryotic Genome Annotation Pipeline (PGAP) (https://www.ncbi.nlm.nih.gov/genome/annotation_prok/) (10). The genome was screened for the presence of putative bacteriocin gene clusters and secondary metabolite biosynthetic gene clusters using BAGEL4 (11). ResFinder v4.1 (12), PathogenFinder v1.1 (13), and RAST FIGfams v70 (14) were used to predict antibiotic resistance genes, pathogenic gene clusters, and identify functional biosynthetic genes, respectively. Default parameters were used for all bioinformatic analyses except otherwise noted.

The *de novo* assembly resulted in an estimated genome size of 3,563,334 bp with 249 contigs (largest contig 76,185 bp), 38.44% G + C content, N_50_ value of 24,301  bp, L_50_ value of 47, and 293.7× genome coverage. PGAP (10) revealed a total of 3,271 genes including 3,182 coding DNA sequences, 35 pseudogenes, 76 tRNAs, 10 rRNAs, and 3 noncoding RNAs. RAST v.2.0 (14) predicted 329 subsystems and 1,551 protein-coding genes involved in putative functional categories, such as biosynthesis of riboflabin, biotin, lipoic acid, folate, pterines, etc. BAGEL4 (11) detected alveicin_B_bacteriocintoxin and colicin-E6 in the genome. No antibiotic resistance genes and immune evasion genes were detected in the genome using ResFinder v4.1 (12). However, PathogenFinder v1.1 (13) predicted CR112 as possibly a human pathogen (probability of being a human pathogen: 0.669, input proteome coverage: 1.32%, matched pathogenic families: 29, and matched not pathogenic families: 12). Further studies are necessary for its safe use as a fish probiotic.

## Data Availability

The whole-genome shotgun project of *Proteus faecis* CR112 has been deposited at GenBank under the accession number JARZHJ000000000. The associated BioProject and BioSample accession numbers are PRJNA958451 and SAMN34300307, respectively. The raw data are available from the Sequence Read Archive (SRA) under accession number SRX20054457.
